# The relationship between dysphagia and frailty among Chinese hospitalized older patients: a serial mediation model through self-perceived oral health and self-reported nutritional status

**DOI:** 10.1186/s12877-024-04684-0

**Published:** 2024-01-29

**Authors:** Jianjiao Yu, Huolan Zhu, Yulian Zhang, Dan Wang, Hua Guo, Xiaomei Liu, Jin Lai, Huiying Zhang, Huanhuan Xu, Bingyue Bai

**Affiliations:** 1 School of Nursing, Xi’an Jiaotong University, 76 Yanta West Road, 710061 Xi’an, China; 2 Department of Geriatrics, Shaanxi Provincial People’s Hospital, 256 Youyi West Road, 710068 Xi’an, China; 3 Shaanxi Provincial Clinical Research Center for Geriatric Medicine, Shaanxi Provincial People’s Hospital, 256 Youyi West Road, 710068 Xi’an, China; 4 Director’s Office, Shaanxi Provincial People’s Hospital, 256 Youyi West Road, 710068 Xi’an, China; 5 Department of Nursing, Shaanxi Provincial People’s Hospital, 256 Youyi West Road, 710068 Xi’an, China; 6 School of Nursing, Shaanxi University of Chinese Medicine, Xixian Road, 712046 Xi’an, China; 7 School of Nursing, Yan’an University, 580 Shengdi Road, 716000 Yan’an, China

**Keywords:** Frailty, Dysphagia, Self-perceived oral health, Self-reported nutritional status, Hospitalized older patients

## Abstract

**Background:**

Frailty contributes to adverse outcomes in older adults and places a heavy burden on healthcare resources. Dysphagia is associated with frailty, but the mechanisms by which dysphagia affects frailty in older adults are unclear. This study aimed to investigate a serial mediating effect of self-perceived oral health and self-reported nutritional status in the relationship between dysphagia and frailty among hospitalized older patients in China.

**Methods:**

This cross-sectional study included 1200 patients aged ≥ 65 years in the Department of Geriatrics, Shaanxi Provincial People’s Hospital. A structured face-to-face interview was used to survey the following questionnaires: General Information Questionnaire, Tilburg Frailty Indicators (TFI), Eating Assessment Tool-10 (EAT-10), 30mL Water Swallow Test (WST), Geriatric Oral Health Assessment Index (GOHAI), and Short-Form Mini-Nutritional Assessment (MNA-SF). A total of 980 participants with complete data were included in the analysis. Statistical analysis was performed using SPSS 26.0 and Amos 28.0 software. Spearman’s correlation analysis was used for correlation analysis of study variables. The results of the multivariate linear regression analysis for frailty were used as covariates in the mediation analysis, and the structural equation model (SEM) was used to analyze the mediating effects among the study variables.

**Results:**

Dysphagia, self-perceived oral health, self-reported nutritional status, and frailty were significantly correlated (*P*<0.001). Dysphagia was found to directly affect frailty (*β* = 0.161, 95%CI = 0.089 to 0.235) and through three significant mediation pathways: (1) the path through self-perceived oral health (*β* = 0.169, 95%CI = 0.120 to 0.221), accounting for 36.98% of the total effect; (2) the path through self-reported nutritional status (*β* = 0.050, 95%CI = 0.023 to 0.082), accounting for 10.94% of the total effect; (3) the path through self-perceived oral health and self-reported nutritional status (*β* = 0.077, 95%CI = 0.058 to 0.102), accounting for 16.85% of the total effect. The total mediation effect was 64.77%.

**Conclusions:**

This study indicated that dysphagia was significantly associated with frailty. Self-perceived oral health and self-reported nutritional status were serial mediators of this relationship. Improving the oral health and nutritional status of hospitalized older patients may prevent or delay the frailty caused by dysphagia.

**Supplementary Information:**

The online version contains supplementary material available at 10.1186/s12877-024-04684-0.

## Introduction

The global aging situation is becoming increasingly critical [1]. Frailty is the greatest challenge facing older adults, a state of age-related decline in physiological reserve, reduced ability to maintain physiological balance, and increased vulnerability to adverse health events [2, 3]. A meta-analysis showed that the prevalence of frailty was 29.8% in hospitals, and was lowest in the community (13.3%) [4]. The prevalence of frailty in hospitalized older adults was higher than that in community-dwelling older adults [4–6]. Since the Corona Virus Disease 2019 (COVID-19) outbreak has put a lot of pressure on people’s health, and although the COVID-19 pandemic has been regularly for prevention and control, it still increases the risk of frailty in older adults [7]. Frailty affects the health of older adults, increases healthcare utilization, and also increases the risk of serious COVID-19 [3, 4, 8, 9]. However, frailty in older adults is potentially reversible [10, 11]. A growing number of studies have focused on risk factors of frailty in older adults. Notably, dysphagia can be consider a risk factor for frailty [12, 13]. Although dysphagia has been shown to be associated with frailty in older adults, its effect on frailty remains unclear.

In addition to direct effect of dysphagia on frailty, research into the pathways involved in this relationship is also very meaningful. During the aging process, both the physiological functions of older adults deteriorate, leading to a range of oral problems. Oral health negatively correlated with frailty in hospitalized older patients [14]. Fewer teeth and poorer oral function were associated with frailty [15]. Meanwhile, previous studies have found a significant correlation between dysphagia and oral health [16]. Considering the association of oral health with dysphagia and frailty, dysphagia may cause poor oral health, which may lead to frailty. Thus it is possible that dysphagia contributes to frailty through poor oral health.

Nutritional status is considered a key contributor to the pathophysiology of frailty [17]. A Chinese study found that hospitalized older patients with good nutrition were less likely to develop frailty [18]. Furthermore, many studies reported that nutritional status was negatively associated with dysphagia [13, 19, 20]. Given the link between nutritional status, dysphagia and frailty, we assume that nutritional status may mediate the relationship between dysphagia and frailty. Meanwhile, preliminary evidence suggested that the relationship between oral health and frailty could be achieved through a nutritional pathway [21]. Therefore, we hypothesized that oral health may be a prerequisite for nutritional status. From the above analysis, oral health and nutritional status may serially mediate the association between dysphagia and frailty, i.e. the mediators oral health and nutritional status exhibit sequential characteristics, forming a mediation chain through which dysphagia has an indirect effect on frailty [22].

**Fig. 1:**
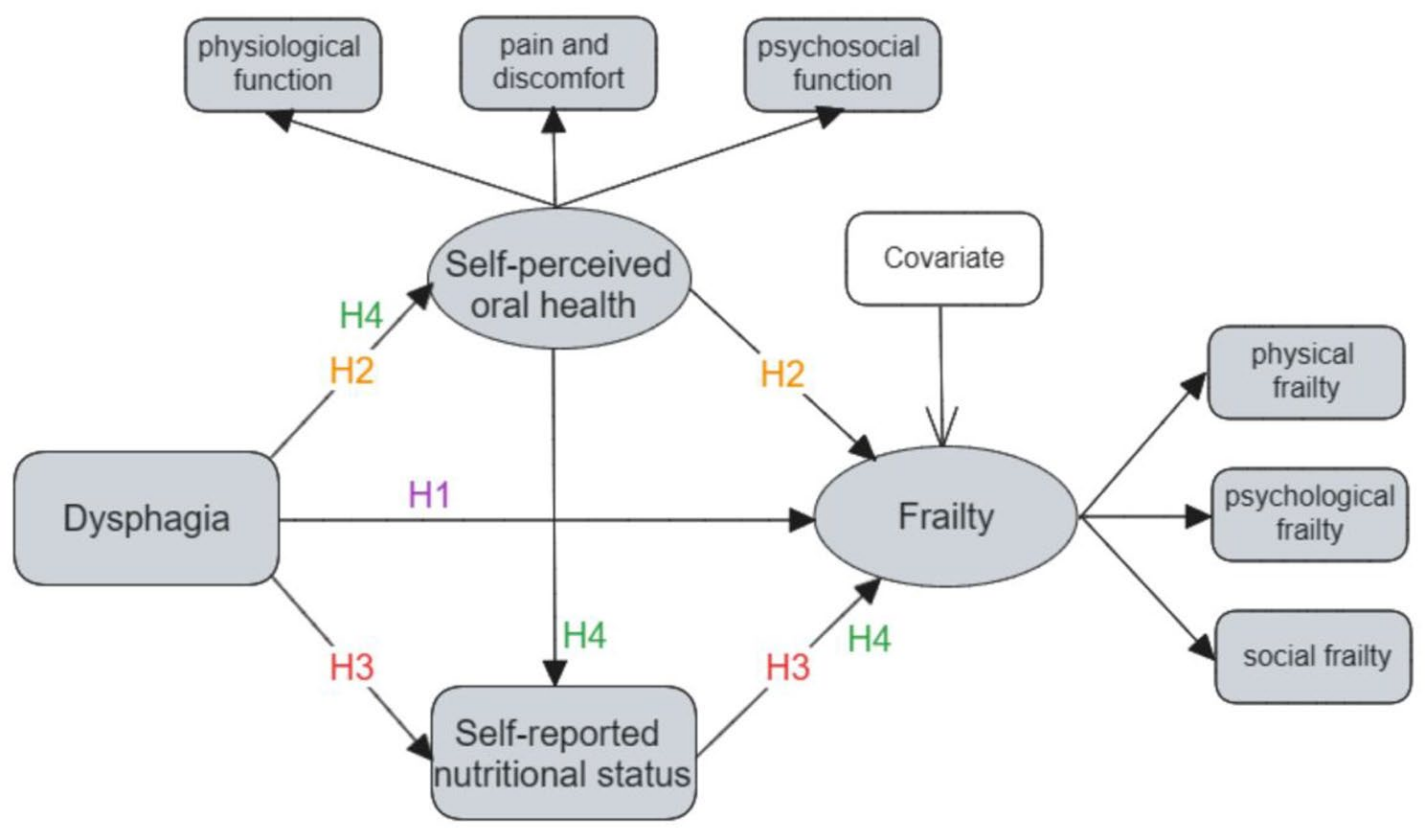
Hypothesized model for this study

Frailty and dysphagia are common problems in older adults and need to be concerned. However, there are few studies on the relationship between dysphagia and frailty, and further in-depth studies on the correlation between the two are needed. The purpose of the present study was to explore the serial mediating effect of self-perceived oral health and self-reported nutritional status in the relationship between dysphagia and frailty. We proposed the following four hypotheses and the hypothesized model (shown in Fig. 1) as follows:

• Hypothesis 1 (H1). Dysphagia is significantly associated with frailty in Chinese hospitalized older patients;
• Hypothesis 2 (H2). Self-perceived oral health mediates the association between dysphagia and frailty;
• Hypothesis 3 (H3). Self-reported nutritional status mediates the association between dysphagia and frailty;
• Hypothesis 4 (H4). Self-perceived oral health and self-reported nutritional status play a serial-mediation role in the relationship between dysphagia and frailty.

## Methods

### Study design and participants

A cross-sectional study with structured face-to-face interview was conducted in the Department of Geriatrics, Shaanxi Provincial People’s Hospital from May 2022 to December 2022, using convenience sampling. Shaanxi Provincial People’s Hospital, located in the urban area of Xi’an City, Shaanxi Province, Northwest China, is a large-scale comprehensive public hospital directly under the provincial government, integrating medical care, emergency care, and rehabilitation.

Inclusion criteria were: (1) age ≥ 65 years; (2) stable condition: in the judgment of the patient’s attending physician, the patient is not currently in an acute or critical condition or in need of resuscitation; (3) able to take food orally; (4) clear consciousness, verbal communication ability, cognitive judgment ability: the patient is clearly conscious, has no history of physician-diagnosed dementia, psychiatric or serious somatic diseases, and has no communication deficits, as determined by review of the patient’s medical record. Individuals were excluded if they had severe difficulty opening their mouths, oral surgery, or trauma.

The sample size was calculated using the Westland-Structural Equation Model (SEM) Sample Size Calculator [23]. Based on expert recommendations, we set the minimum effect to detect at 0–0.12, significance at 0.05, and power at 0.8. According to our hypothesized model (assuming all 10 general information as covariates), the number of latent variables and indicators variables in our study was 2 and 18, respectively. Therefore, the minimum required sample size for this study was 591. Taking into account the loss and refusal of interviews, a total of 1200 questionnaires were finally distributed.

### Measures

#### General information questionnaire

To obtain reliable results, general information related to frailty needs to be controlled, and after reviewing the literature [3, 24], the following general information questionnaire was developed: gender, age, marital status, educational level, physical exercise (judged to be ≥ 3 times per week for ≥ 30 min each time, including jogging, eight-dan brocade, tai chi, balance training, etc.), sleep disorders (include inability to fall asleep within 30 min, waking up easily at night, dreaminess, snoring, and waking up early), constipation (defined as bowel movements ≤ 3 times per week in the past 6 months), comorbidity (referred to having ≥ 2 kinds of chronic diseases, including hypertension, heart disease, stroke, diabetes, tumors, etc.), polypharmacy (defined as taking ≥ 5 types of medications per day, including prescription, over-the-counter, and Chinese herbal medications), and denture use.

#### Frailty

Frailty was assessed with the Tilburg Frailty Indicators (TFI) [25]. It is a self-report scale consisting of three dimensions of physical frailty (8 items), social frailty (3 items), and psychological frailty (4 items), for a total of 15 items. The scale adopts a dichotomous scoring method, with the total score ranging from 0 to 15. And a score of ≥ 5 indicates frailty in older adults. The Chinese version of TFI had good reliability and validity, and can be used as a frailty assessment tool in elderly inpatients [26]. In this study, the Cronbach’s alpha was 0.748.

### *Dysphagia*

Dysphagia was examined by the Eating Assessment Tool-10 (EAT-10) [27] and the 30mL Water Swallow Test (WST) [28]. In the present study, dysphagia was considered to be present if the results of both tools indicated dysphagia.

The EAT-10 is a 10-item self-report scale. In 2013, the Chinese expert consensus on rehabilitation assessment and treatment of dysphagia translated it and recommended its use [29]. The EAT-10 consists of 10 questions pointed from 0 to 4, with a total score of 0–40. For each question, 0 indicates “no problem”, while 4 indicates “severe problem”, with higher score suggesting more severe swallowing problem. A cut-off value of 3 was recommended as the best cut-off value for the EAT-10 [30]. The Cronbach’s alpha was 0.907 in this sample.

The WST requires patients to drink 30mL of water at a normal rate in an upright position and observes the time required and whether choking occurs. Based on the observation results, patients are classified as Grade I–IV. Normal: Grade I (< 5 s); suspicious: Grade I (≥ 5 s) or Grade II; abnormal: Grade III–V. Suspicious and abnormal were treated as dysphagia in our study.

#### Oral health

The Geriatric Oral Health Assessment Index (GOHAI) was employed to evaluate self-perceived oral health. It includes three dimensions of physiological function (4 items), pain and discomfort (3 items), and psychosocial function (5 items), for a total of 12 items. The Likert 5-point scoring method is used. With a total score of 12–60, oral health-related quality of life (OHRQoL) is divided into three levels: high (57–60), medium (51–56), and low (≤ 50) [31, 32]. The higher the score of GOHAI, the better the OHRQoL. Chinese scholars found that the Chinese version of GOHAI had good reliability and validity [32]. The Cronbach’s alpha for this sample was 0.863.

#### Nutritional status

The Short-Form Mini-Nutritional Assessment (MNA-SF) was used to assess self-reported nutritional status [33]. The MNA-SF is composed of 6 items: (1) Ask the patient about his/her weight loss in the past 3 months. (2) Body mass index (BMI) = weight/height^2^ (kg/m^2^), height and weight are measured on a scale in the department, with shoes off and only a gown on. If the patient is unable to get out of bed, the calf circumference is measured. (3) Ask the patient if he/she has suffered any trauma or acute illness in the past 3 months. (4) Mobility: ask the patient if he/she is bedridden or wheelchair-bound for a long period of time, and if he/she goes out to do activities. (5) Spiritual and psychological: combine the answers from the patient, his/her family and his/her doctor to make a comprehensive judgment.6) Whether he/she has eaten less food due to loss of appetite, dyspepsia, difficulty in chewing or swallowing in the past 3 months: severe reduction in eating is defined as more than 75% reduction in eating, moderate reduction is defined as 10–75%, and no change is defined as a change in eating within ± 10%. The total score ranges from 0 to 14. The assessment results are classified as normal nutritional status (12–14), risk of malnutrition (8–11), and malnutrition (0–7). The higher the score, the better the nutritional status. Chinese scholars found that the sensitivity and specificity of MNA-SF were high, and it can be used for early assessment and screening of nutritional status in Chinese older adults [34]. The Cronbach’s alpha was 0.686.

### Data collection

The researcher conducted a pilot study on 35 older patients at the Department of Geriatrics, Shaanxi Provincial People’s Hospital in May 2022. The results of the pilot study showed that: the reliability of the TFI, EAT-10, GOHAI, and MNA-SF was good; the patients’ cooperation was high; and the questionnaires were operable and took an average of 15 min to complete, which was within the patients’ acceptable range. Based on the results of the pilot study and patients’ feedback, the researcher revised the questionnaire and the study protocol.

The data collectors for this study were five postgraduate medical students and eight medical staff, who were received uniform training before the start of the study. Age-eligible patients were first screened in a computerized system, and then we further evaluated our inclusion and exclusion criteria on the wards. After obtaining informed consent, investigators collected questionnaire data through face-to-face, one-on-one personal interviews on the ward, during which the investigator read each question aloud and objectively marked the participant’s answers on the questionnaire. At the end of the survey, we removed questionnaires with incomplete answers.

### Statistical analysis

EpiData 3.1 software was used to establish the database, and data were entered independently by two researchers. Data were analyzed using SPSS 26.0 and Amos 28.0 software. A *P*-value < 0.05 (two-tailed) was considered statistically significant.

We analyzed descriptive statistics for general information and key study variables. As the distributions of age, TFI score, GOHAI score, and MNA-SF score were skewed, these continuous variables were described using the median (interquartile range [IQR]). Categorical variables were expressed as frequency (percentage). To meet the assumptions of the mediation model [35], Spearman’s correlation analysis was used to examine the general relationships between the four variables: dysphagia, self-perceived oral health, self-reported nutritional status, and frailty. Multivariate linear regression analyses were used to determine the influencing factors of frailty, which were used as covariates in mediation analysis.

An SEM was constructed using maximum-likelihood estimator in Amos 28.0 software to test the hypothesized model. In the mediation model, the relationship between two variables was expressed as path standardized coefficient (β); the effect of mediation was expressed as standardized effect, with the direct effect equal to the path standardized coefficient from dysphagia to frailty, and indirect effects of dysphagia on frailty via the mediators (self-perceived oral health or self-reported nutritional status) were equal to the multiplication of each of the path standardized coefficients. The total effect is equal to the direct effect plus the indirect effect. The mediating effect is the value of the direct or indirect effect as a percentage of the total effect. Hayes’ bias-corrected bootstrap [36] was used to examine the total, direct, and indirect effects in the model. We set the bootstrap confidence interval (CI) at 95%, and the number of bootstrap samples was 5,000. If the 95%CI for the indirect effects did not contain zero, and *P*-values < 0.05, it indicated that the mediating effect was significant. Because SEM samples greater than 200 or more easily caused by the *χ*^2^ value is too large resulting in poor fit, so the Bollen-Stine Bootstrap correction model fit was used for the model [37]. The model fit indices included the Bollen-Stine chi-square value/degree of freedom (*χ*^2^/*df*, < 5.00), goodness-of-ft index (GFI, >0.90), adjusted goodness-of-ft index (AGFI, >0.90), comparative fit index (CFI, >0.90), Tucker-Lewis index (TLI, >0.90), and root mean square error approximation (RMSEA, < 0.08) [38].

### Common method biases

In this study, Harman’s single-factor test for common method bias was used [39]. The result of the unrotated factors showed that the eigenvalues of 11 factors were greater than 1, suggesting that more than one factor underlies the data. The variance explained by the first factor was 24.05%, which was less than the threshold of 40%. The above analysis indicated that there were no serious common method biases in our study.

### Ethical aspects

This study protocol was reviewed and approved by the Ethics Committee of Shaanxi Provincial People’s Hospital (Xi’an, Shaanxi Province, China), and the approval number of the Ethics Committee was 2021R001. Written informed consent was obtained from all participants or their legal guardians at recruitment. In this study, all data collected, processed, and analyzed were anonymous and used only for the research purposes.

## Results

### Characteristics of the participants

A total of 1200 questionnaires were finally distributed, of which 980 valid questionnaires were included in our study, for a response rate of 81.7%. Of the 980 participants, a higher proportion of men (52.1%) than women (47.9%) participated in the survey. The age range was 65–99 years with a median of 72 years (IQR: 68–78). The vast majority of patients were married (87.0%). Among educational level, high school accounted for the highest percentage (30.7%), and the lowest was primary school or below (13.3%). Physical exercise, sleep disorders, constipation, comorbidy, polypharmacy, and denture use accounted for 49.9%, 45.4%, 34.5%, 78.8%, 50.3%, and 35.4% respectively. The prevalence of frailty was 49.1%, and dysphagia was 14.7%. The scores of frailty, self-perceived oral health, and self-reported nutritional status were 4 (3–7), 54 (48–57), and 12 (10–13), respectively (Table 1).

**Table 1:** Description of the sample

Characteristics	N (%)	Median (IQR)
Observations	980	
Gender		
Men	511 (52.1)	
Women	469 (47.9)	
Age, years		72 (68–78)
Marital status		
Married	853 (87.0)	
Unmarried/divorced/widowed	127 (13.0)	
Educational level		
College or above	276 (28.2)	
High school	301 (30.7)	
Middle school	273 (27.9)	
Primary school or below	130 (13.3)	
Physical exercise		
No	491 (50.1)	
Yes	489 (49.9)	
Sleep disorders		
No	535 (54.6)	
Yes	445 (45.4)	
Constipation		
No	642 (65.5)	
Yes	338 (34.5)	
Comorbidity		
No	208 (21.2)	
Yes	772 (78.8)	
Polypharmacy		
No	487 (49.7)	
Yes	493 (50.3)	
Denture use		
No	633 (64.6)	
Yes	347 (35.4)	
Frailty		
No	499 (50.9)	
Yes	481 (49.1)	
Frailty, score		4 (3–7)
Dysphagia		
No	836 (85.3)	
Yes	144 (14.7)	
Self-perceived oral health, score		54 (48–57)
Self-reported nutritional status, score		12 (10–13)

Note: N (%), frequency (percentage); IQR, interquartile range

### Correlation between dysphagia, self-perceived oral health, self-reported nutritional status and frailty

The correlation matrix for the key study variables was shown in Table 2. First, dysphagia was significantly positively correlated with frailty (*r* = 0.421). Second, self-perceived oral health and self-reported nutritional status were significantly negatively correlated with frailty, with correlation coefficients of 0.561 and 0.556, respectively. Third, self-perceived oral health may promote the development of self-reported nutritional status (*r* = 0.474). Finally, dysphagia was negatively correlated with self-perceived oral health and self-reported nutritional status, with correlation coefficients of 0.432 and 0.323, respectively. The results of the correlation analysis suggested that there may be some mediating effects between the above variables.

**Table 2:** Correlation between dysphagia, self-perceived oral health, self-reported nutritional status and frailty

Variables	1	2	3	4
1.Frailty	1			
2.Dysphagia	0.421*	1		
3.Self-perceived Oral health	-0.561*	-0.432*	1	
4.Self-reported Nutritional status	-0.556*	-0.323*	0.474*	1

Note: *P-value <0.001 (two-tailed)

### Mediating effect analysis

**Fig. 2:**
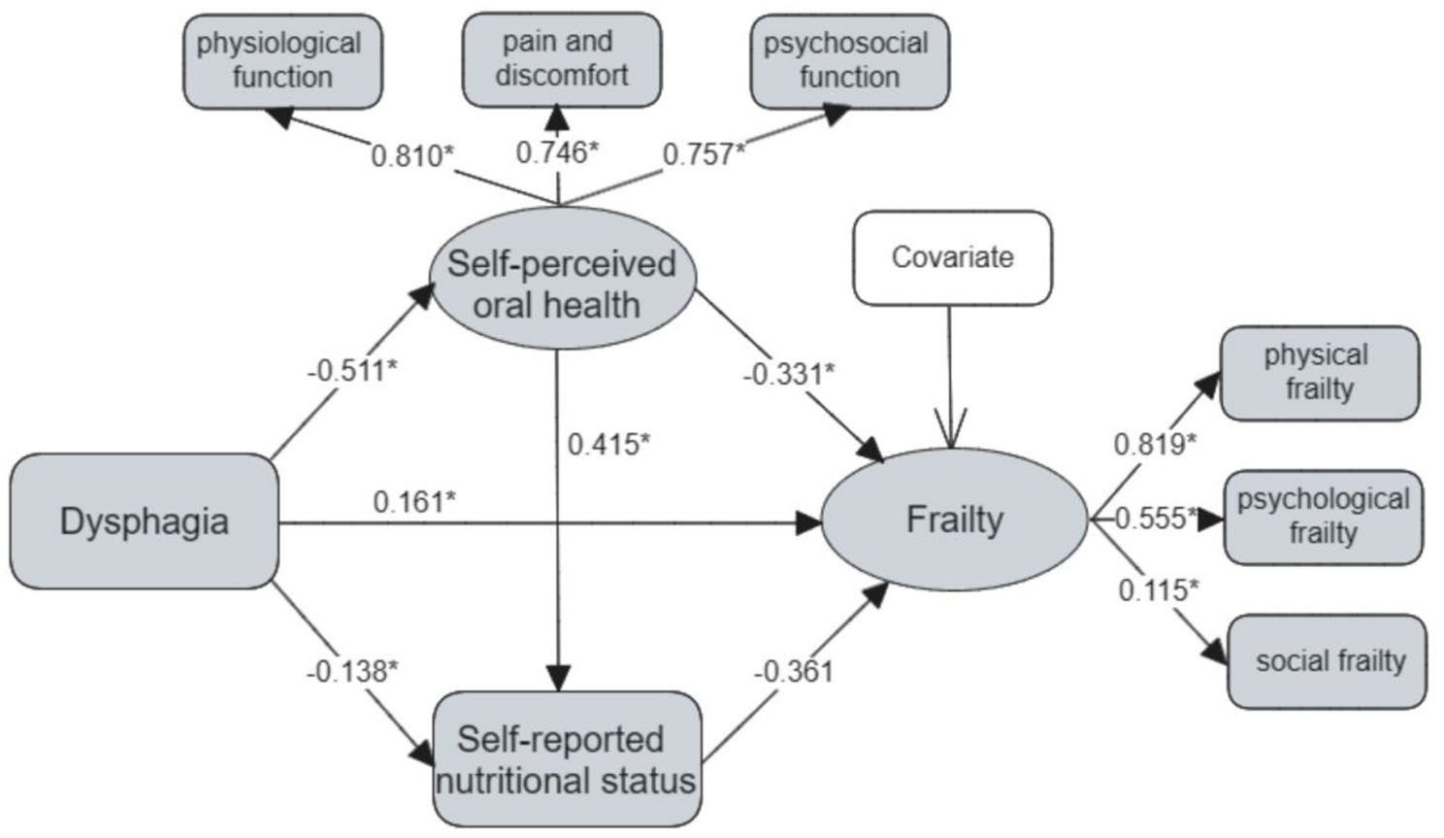
A serial mediation model of the association between dysphagia and frailty. Note: *P-value<0.001 (two-tailed). Covariates for frailty were age, marital status, educational level, physical exercise, sleep disorders, constipation, comorbidity, and polypharmacy

The model (Fig. 2) controlled eight variables for frailty: age, marital status, educational level, physical exercise, sleep disorders, constipation, comorbidity, and polypharmacy according to the result of the multivariate linear regression analysis of frailty (see additional file 1). The proposed mediation model showed acceptable goodness of fit (Bollen-Stine *χ*^2^/df = 1.057, GFI = 0.981, AGFI = 1.152, CFI = 0.999, TLI = 0.998, and RMSEA = 0.074).

Figure 2; Table 3 presented the results of the path analysis of the variables. The path standardized coefficients (β) showed that all relationships in the model were significantly correlated. Specifically, the effect of dysphagia on frailty was significant (β = 0.161, SE = 0.037, *P*<0.001), indicating that dysphagia was associated with frailty in hospitalized older patients. Dysphagia was negatively associated with self-perceived oral health (β=-0.511, SE = 0.032, *P*<0.001) and self-reported nutritional status (β=-0.138, SE = 0.039, *P*<0.001), suggesting that hospitalized older patients with dysphagia are more likely to have oral health problems and malnutrition. Similarly, self-perceived oral health had a positive effect on self-reported nutritional status (β = 0.415, SE = 0.036, *P*<0.001). In addition, the association between self-perceived oral health (β=-0.331, SE = 0.043, *P*<0.001), self-reported nutritional status (β=-0.361, SE = 0.037, *P*<0.001) and frailty was negative.

**Table 3:** Path analysis of the model

Path	Standardized Coefficient	SE	*P*-value	Bootstrapping 95%CI
Dysphagia→Frailty	0.161	0.037	<0.001	(0.089 to 0.235)
Dysphagia→Self-perceived oral health	-0.511	0.032	<0.001	(-0.570 to -0.446)
Self-perceived oral health→Frailty	-0.331	0.043	<0.001	(-0.411 to -0.399)
Dysphagia→Self-reported nutritional status	-0.138	0.039	<0.001	(-0.213 to -0.064)
Self-reported nutritional status→Frailty	-0.361	0.037	<0.001	(-0.431 to -0.285)
Self-perceived oral health→Self-reported nutritional status	0.415	0.036	<0.001	(0.342 to 0.487)

Note: SE, standard error; CI, confidence interval

Table 4 presented the bootstrap analysis results of the mediating effect of self-perceived oral health and self-reported nutritional status in the relationship between dysphagia and frailty. The 95%CIs of the total effect, direct effect and indirect effects did not contain zero. Therefore, the association between dysphagia and frailty was serially mediated by these two mediators. The standardized effect of dysphagia on frailty through self-perceived oral health was 0.169, with a mediating effect of 36.98%. The standardized effect of dysphagia on frailty through self-reported nutritional status was 0.050, with a mediating effect of 10.94%. The standardized effect of dysphagia on frailty through self-perceived oral health and self-reported nutritional status was 0.077, with a mediating effect of 16.85%. The total mediating effect of dysphagia on frailty was 64.77%, which was stronger than the direct effect of dysphagia on frailty.

**Table 4:** Bootstrap analysis of Total, direct, and indirect effects

Model pathways	Standardized effect	SE	Bootstrapping 95%CI	Mediating effect
Total effect				
dysphagia→frailty	0.457*	0.033	(0.389 to 0.519)	100%
Direct effect				
dysphagia→frailty	0.161*	0.037	(0.089 to 0.235)	35.23%
Total indirect effect	0.296*	0.028	(0.244 to 0.351)	64.77%
dysphagia→self-perceived oral health→frailty	0.169*	0.025	(0.120 to 0.221)	36.98%
dysphagia→self-reported nutritional status→frailty	0.050*	0.015	(0.023 to 0.082)	10.94%
dysphagia→self perceived oral health→self- reported nutritional status→frailty	0.077*	0.011	(0.058 to 0.102)	16.85%

Note: SE, standard error; CI, confidence interval. *P-value<0.001 (two-tailed)

## Discussion

The prevalence of frailty among hospitalized older patients in our study was 49.1%, which was higher than in a previous study (29.8% in hospitals) [4]. This may be due to an increase in the prevalence of frailty in older adults as a result of the COVID-19 pandemic [40]. The current study mainly found that dysphagia was positively associated with frailty directly, and also indirectly through self-perceived oral health and self-reported nutritional status.

### The direct effect of dysphagia on frailty

This study confirmed that dysphagia had a direct effect on frailty in hospitalized older patients with an effect size of 35.23%, validating H1. The direct effect of dysphagia on frailty was consistent with previous studies [12, 13, 41, 42]. With age, atrophy of swallowing muscles and reduced elasticity of connective tissues may cause impaired strength and ductility of swallowing, making older adults vulnerable to dysphagia [43]. Dysphagia can lead to inadequate energy and nutrient intake in older patients, resulting in weight loss, decreased muscle strength, easy fatigue, and other indicators of frailty [12]. In addition, aspiration, a complication of dysphagia, is also an important trigger of frailty. When dysphagia exists in older adults, respiratory and swallowing functions are uncoordinated, making choking and aspiration easy, allowing oral colonizing bacteria to enter the lungs and develop pulmonary inflammation [44]. While inflammatory biomarkers are important emerging markers of frailty [45]. Research has shown that frailty prevention strategies, including swallowing training, may be helpful for older adults [42]. However, in our study, the total mediating effect was 64.77%, which was stronger than the direct effect, revealing that our mediators were crucial in explaining the association between dysphagia and frailty.

### The mediation effect of self-perceived oral health and self-reported nutritional status

Our findings showed that self-perceived oral health mediated the relationship between dysphagia and frailty with a mediating effect of 36.98%. Thus, H2 was supported: hospitalized older patients with dysphagia were more likely to suffer from frailty due to poor oral health. With the increase of age, the basic structure and physiological function of the oral cavity undergoes degenerative changes that can lead to a variety of oral health problems. Poor oral health may be linked to frailty through four mechanisms: malnutrition, chronic inflammation, psychosocial factors, and medication effects [14]. The worse the oral health, the more likely older patients are to be frail. Frailty is not only affected by oral health, but also has a negative impact on oral health, forming a vicious cycle [46]. Dysphagia may result in poor bolus clearance, oral residue and poor saliva management in patients, which are likely to contribute to poorer oral health [47]. Oral hygiene interventions could effectively improve the oral health of patients with dysphagia [47]. Our study found that self-perceived oral health played the strongest mediating role in the association between dysphagia and frailty, suggesting the importance of oral health management for older patients with dysphagia.

Our results showed that self-repored nutritional status was a mediator in the association between dysphagia and frailty, with a mediating effect of 10.94%. Therefore, H3 was tested that hospitalized older patients with dysphagia were more inclined to become frailty because of malnutrition. In the first part of this mediation process, dysphagia was negatively associated with self-reported nutritional status, which was consistent with previous studies [13, 20]. Dysphagia can lead to malnutrition in older adults with limited food intake and poor diet quality [48]. At the same time, nutritional supplementation could counteract sarcopenic dysphagia [49]. In the second part of this mediation process, self-reported nutritional status was negatively associated with frailty, similar to prior research [18]. Malnourished people generally lack sufficient protein, vitamins (B6, B12, D, etc.) and minerals, which may cause loss of muscle mass and muscle strength, thus promoting the process of frailty in older adults [50]. Nutritional deficiencies can also reduce immune function, increasing the risk of infection and complications and exacerbating frailty. The use of tailored foods and supplements, combined with dietary education, has improved frailty in older adults [51]. The Mediterranean diet is currently considered by international guidelines to be an appropriate dietary pattern for frailty [52].

### The serial multiple mediation model of self-perceived oral health and self-reported nutritional status

The present study also verified that self-perceived oral health and self-reported nutritional status played a serial mediating role in the effect of dysphagia on frailty in hospitalized older patients, confirming H4. The mediation effect was 16.85%. The results of this study demonstrated that dysphagia first had a negative correlation with self-perceived oral health and then caused a decline in self-reported nutritional status, which in turn was associated with frailty. Poor oral health and malnutrition were associated with dysphagia. Besides, poor oral health has been identified as a risk factor for malnutrition [53, 54]. Missing teeth, dental caries, dysgeusia, periodontal disease, etc. reduce chewing function of older adults, decrease the intake of food and nutrients, aggravate the gastrointestinal burden, resulting in malnutrition [55, 56]. Older adults with dysphagia often experience serious oral health problems, which is a risk factor for poor nutritional status and ultimately increases the likelihood of frailty.

### Implications for practice

The major contribution of our study was the finding that dysphagia could both directly and indirectly affect frailty via self-perceived oral health and self-reported nutritional status in hospitalized older patients. Therefore, patients with dysphagia could prevent and delay frailty by improving oral health and nutritional status. First, a simple and effective screening tool for dysphagia, such as the EAT-10 combined with the WST [29], can be used in the clinic, and older adults with a problematic initial screening can be further examined for confirmation and prompt swallowing function training. Second, we should be concerned about the oral health of patients with dysphagia. Provide timely oral health education and oral training [57, 58]. Last but not least, it is important to enhance the assessment of nutritional status of older adults with dysphagia, along with dietary interventions and health education [49, 59]. The current findings had practical implications for the management of frailty.

### Limitations and future research

Our study has several limitations. First, the cross-sectional nature hindered the definitive establishment of causality, and future research should include longitudinal or experimental studies. Second, only one hospital was selected and convenience sampling was used for this study, and the exclusion of some patients with severe mouth-opening difficulties that overestimated oral health status, which may limit the generalizability of our findings. Expansion of the study population is recommended. Third, except for dysphagia, where subjective and objective measures were used, all other variables were self-reported measures with potentially biased values. Because of the scale question settings, no relevant physical examination was performed, and the health status of older adults is likely to be overestimated to the extent that TFI scores are low, GOHAI scores are high, and MNA-SF scores are high. The use objective indicators could overcome the common problems of questionnaires. Finally, our study only tested the specific mediating pathways described above, without considering other possible mediators, nor did it consider whether there were moderators that might influence the role of oral health and nutritional status in the relationship between dysphagia and frailty. Other potential mechanisms should be further explored.

## Conclusion

In conclusion, dysphagia had a significant positive effect on frailty in Chinese hospitalized older patients, with self-perceived oral health and self-reported nutritional status playing partial mediating roles. Considering the relationship between dysphagia and frailty via self-perceived oral health and self-reported nutritional status, it is possible that hospitalized older patients with good oral health and nutritional status may be better able to cope with dysphagia and thus delay the onset of frailty. The results of this study provide directions for further research to understand the relationship between dysphagia and frailty in older adults, which may inform future frailty management.

### Electronic supplementary material

Below is the link to the electronic supplementary material.

Supplementary Material 1

## Data Availability

The data that support the findings of this study are available from the corresponding author on request.

## Abbreviations

TFI: Tilburg Frailty Indicators
EAT-10: Eating Assessment Tool-10
WST: 30mL Water Swallow Test
GOHAI: Geriatric Oral Health Assessment Index
MNA-SF: Short-Form Mini-Nutritional Assessment
SEM: Structural equation model
COVID-19: Corona Virus Disease 2019
BMI: Body mass index
IQR: Interquartile range
β: Standardized coefficients
CI: Confidence interval
*χ*^2^/df: Chi-square value/degree of freedom
GFI: Goodness-of-ft index
AGFI: Adjusted goodness-of-ft index
CFI: Comparative fit index
TLI: Tucker-Lewis index
RMSEA: Root mean square error of approximation
SE: Standard error
